# Draft Genome Sequence of *Clostridium difficile* Belonging to Ribotype 018 and Sequence Type 17

**DOI:** 10.1128/genomeA.00907-16

**Published:** 2016-09-01

**Authors:** E. Riccobono, V. Di Pilato, N. Della Malva, S. Meini, F. Ciraolo, F. Torricelli, G. M. Rossolini

**Affiliations:** a Department of Experimental and Clinical Medicine, University of Florence, Careggi University Hospital, Florence, Italy; b Department of Surgery and Translational Medicine, University of Florence, Florence, Italy; c Internal Medicine Unit, Santa Maria Annunziata Hospital, Florence, Italy; d Office of the Chief Medical Officer, Santa Maria Annunziata and Serristori Hospitals, Florence, Italy; e Genetic Diagnostic Unit, Florence Careggi University Hospital, Florence, Italy; f Department of Medical Biotechnologies, University of Siena, Santa Maria alle Scotte University Hospital, Siena, Italy; g Clinical Microbiology and Virology Unit, Careggi University Hospital, Florence, Italy; h Don Carlo Gnocchi Foundation, Florence, Italy

## Abstract

*Clostridium difficile*, belonging to ribotype 018 (RT018), is one of the most prevalent genotypes circulating in hospital settings in Italy. Here, we report the draft genome of *C. difficile* CD8-15 belonging to RT018, isolated from a patient with fatal *C. difficile*-associated infection.

## GENOME ANNOUNCEMENT

*Clostridium difficile* infection (CDI) is one of the most common hospital-acquired infections and the leading cause of antibiotic-associated diarrhea and pseudomembranous colitis (1). Increasing CDI incidence rates have been mainly attributed to successful clones belonging to a few predominant ribotypes (RTs), with different distribution in different geographic regions (2, 3).

RT018 has been reported as one of the most prevalent genotypes circulating in hospital settings in Italy (4, 5) and in the Far East (i.e., South Korea and Japan) (6, 7) but has been rarely described in other countries (2, 8). RT018 is highly transmissible and generally shows a multidrug-resistant phenotype. It produces both toxin A and toxin B, but is negative for binary toxin, and is associated with complicated outcomes (4, 7, 9). In this report, we announce the draft genome of *C. difficile* CD8-15 belonging to RT018, which was isolated from a patient admitted to a hospital in Florence (central Italy) in 2015 with fatal *C. difficile*-associated infection evolved as toxic megacolon.

*C. difficile* CD8-15 was grown in 5 ml of thioglicollate broth and incubated under anaerobic conditions for 48 h at 37°C. Genomic DNA was subjected to whole-genome sequencing with the MiSeq platform (Illumina Inc., San Diego, CA, USA) using a 2×300-bp paired-end approach. A total of 2,118,990 reads were generated and then *de novo* assembled using SPAdes version 3.6.1 (10) into 44 scaffolds (largest scaffold = 831,956 bp; *N*_50_ = 235,991 bp; *L*_50_ = 5; average GC = 28.63%), with an estimated genome size of 4,249,791 bp and an average coverage of 50×.

The draft genome was then subjected to the following *in silico* analyses: (i) multilocus sequence typing (MLST; http://pubmlst.org/cdifficile); (ii) toxinotyping (http://www.mf.uni-mb.si/mikro/tox); (iii) detection of antibiotic resistance mechanisms, using the *C. difficile* 630 strain (11). The presence of phage-related sequences and CRISPR-Cas systems were investigated using PHAST (http://phast.wishartlab.com) and CRISPRFinder (http://crispr.u-psud.fr), respectively.

MLST analysis assigned *C. difficile* CD8-15 to sequence type (ST) 17, as previously reported for other *C. difficile* strains belonging to RT018 (12, 13). ST17 and other STs of *C. difficile* belong to clade I (12). Toxinotyping classified *C. difficile* CD8-15 as a nonvariant strain (toxinotype 0) producing toxin A and toxin B but not binary toxin CDT.

*C. difficile* CD8-15 was resistant to rifampin (MIC >32 mg/L), levofloxacin (MIC >32 mg/L), and erythromycin (MIC >256 mg/L) but remained susceptible to clindamycin (MIC 2 mg/L). Sequence analysis revealed the presence of point mutations in the *rpoB* gene (R505K and I548M), which is known to be associated with high-level resistance to rifamycins (14). Mutations were detected also in the quinolone resistance-determining region of *gyrA* (T82I), which is consistent with the fluoroquinolones resistance phenotype. Resistance to erythromycin was likely attributable to the presence of a multidrug efflux system encoded by the *cme* gene (15), while *erm* genes and other genes involved in macrolides resistance (i.e., *mef*, *msr, cfr*) were not detected (9, 16). PHAST identified 3 intact and 5 incomplete prophage regions. CRISPRFinder identified two class I CRISPR-Cas systems.

### Accession number(s).

This whole-genome shotgun project has been deposited at DDBJ/EMBL/GenBank under the accession number LYDP00000000.
